# Does the Dutch trial prove we should “say no” to active surveillance? An in-depth review of the 2025 study on the treatment of esophageal cancer

**DOI:** 10.1016/j.xjon.2025.10.019

**Published:** 2025-10-28

**Authors:** Brian N. Housman, Stephanie Tuminello, Raja Flores

**Affiliations:** a Department of Thoracic Surgery, Icahn School of Medicine at Mount Sinai, New York, NY; b Institute for Translational Epidemiology, Icahn School of Medicine at Mount Sinai, New York, NY

**Keywords:** esophageal cancer, clinical complete response, active surveillance, surgery, SANO trial

**Figure:**
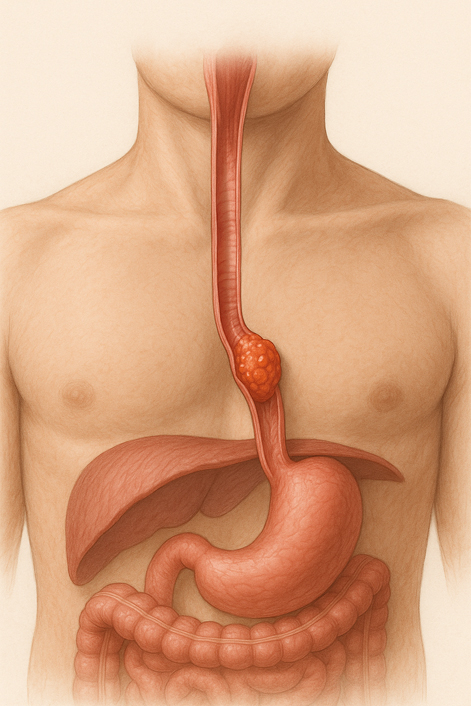
Esophageal cancer.

**Central Message**

Contrary to its conclusions, the SANO trial may have published the strongest condemnation of definitive chemoradiation in the literature and suggests that we should “say no” to active surveillance.

**Perspective**

Active surveillance for esophageal cancer impedes patient safety. Screening technology is limited by high rates of false-negatives and gives a false sense of security. The required clinical complete response is unreliable and does not predict beneficial outcomes and treatment. This review explores the granular detail of the SANO trial and suggests the study's outcomes are different to its conclusions.

“Neoadjuvant Chemoradiotherapy Followed by Active Surveillance versus Standard Surgery for Oesophageal Cancer (SANO Trial): A Multicentre, Stepped-Wedge, Cluster-Randomised, Non-Inferiority, Phase 3 Trial” Was recently published by van der Wilk and colleagues in *Lancet Oncology*.^1^ The goal of the trial was to evaluate the treatment-related outcomes of patients with esophageal cancer who were all candidates for surgical resection. Participants were included if they were 18 years or older, had locally advanced esophageal carcinoma, and who were scheduled for neoadjuvant chemoradiation followed by surgery.

Between 2017 and 221, 1115 patients were screened, of whom 776 were enrolled and underwent neoadjuvant chemoradiation per CROSS (Chemoradiotherapy for Oesophageal Cancer followed by Surgery Study) protocols. After treatment, patients underwent 2 clinical response evaluations (CREs) at 6 and 12 weeks involving endoscopy with endoscopic ultrasound, fine-needle aspiration, bite-on-bite biopsies, and positron emission tomography/computed tomography. After both evaluations, all patients with a “complete clinical response” (CCR) were randomized into treatment groups. CCR was defined as no identifiable malignancy or suspicion of malignancy.

There were 274 patients who achieved CCR. After crossover and patients included from the pre-SANO trial, 111 patients were randomized to the surgery group and 198 to the active surveillance (AS) group.

Overall survival at 2 years—the primary outcome—was found to be 74% in the AS group and 71% in the surgery group, which satisfied the noninferiority benchmark of 15%. The authors concluded that AS is noninferior to surgery and long-term follow-up should be considered as an alternative to esophagectomy for patients with CCR.

To their credit, the authors were among the first to perform a randomized trial comparing 2 populations of surgical candidates. In so doing, they compared patients of similar health and address a common bias caused by clinical asymmetry. Unfortunately, there are a number of other grave concerns that should be addressed and call the results of this study into question.

## Methods

No institutional review board approval was required for this article. There was no patient contact, or need for written consent in the publication of this study.

### CCR is Not Reliable

The validity of the SANO trial balances on a single fulcrum; CREs can detect cancer. Any patient with identifiable disease—or even the suspicion of disease—was immediately removed from the AS group and underwent surgery. If the SANO procedures are not reliable, there is no mechanism to facilitate safe surveillance.

The authors describe that their protocol correctly identified 94% of locoregional regrowth. The number comes from the observation that after 8 CREs, 96 of the 198 patients in the AS group had locoregional failure and 90 were correctly identified. Although the statistic is technically accurate, it is also a misleading commentary on the capabilities of the CRE with the following concerns: (1) It is not clear how the authors determined that only 96 patients had locoregional recurrence. They may be referring to patients with visible tumor on endoscopy, but this is not described. (2) Endoscopic biopsies performed on tumors should accurately diagnose cancer more frequently than 94% of the time. If they do not, it is important to explore what factors limited diagnosis. (3) This is not the sensitivity of the CRE because, (4) without the gold standard of surgical pathology, false-negative results cannot be accurately counted, and sensitivity cannot be calculated. Otherwise, the CRE is only being evaluated against itself, and only in a limited subset of patients with visible tumor.

Fortunately, a false-negative rate of 65% was published in the surgical group (see the Supplemental Table S6 from the SANO trial), which is greater than any in the known literature.^2^ Every patient randomized to surgery was first confirmed to have complete response by the same CRE as patients randomized to AS. Unfortunately, T0 (no identifiable tumor, or a complete pathologic response) was only identified in 36 of 101 patients (35.6%). This means the CRE failed to detect cancer in 65% of cases. Perhaps worse, 38 patients with missed diagnoses had advanced disease (T2 or T3).

It is also noteworthy that the 96 patients with recurrence do not reflect the full term of the study. By the conclusion of the trial, 129 patients (65%) developed some form of recurrence and only 35% of patients in the AS group (n = 69) had “persistent CCR” (see the Supplemental Table S4 from the SANO trial). This mirrors the 65% false-negative rate identified after surgery, and the missed cancers likely resulted in metastatic disease. Of the 129, only 83 patients (64%) made it to surgery, meaning that 46 patients (36%) may have missed their opportunity for resection.

These outcomes include 33 patients (17%) in the AS group who developed distant metastases with negative biopsies, further suggesting primary tumor was missed. Even biopsies that were positive were not always be reliable because 5 patients in the AS group were diagnosed with recurrence but were found to have no identifiable tumor after surgery.

The supplementary materials clarify that of the group of 69 patients (35%) with persistent CCR, 5 died, and 6 refused AS, bringing the actual number of patients with confirmed CCR down to 58 (29%). The remaining patients are reportedly still being followed but are at various stages in their treatment: 20 patients at 30 months, 24 at 36 months, 12 at 48 months, and only 2 patients who stopped surveillance at 5 years, preventing a total accounting of long-term outcomes. The rate of recurrence in the standard surgery group does not appear to be published in either the manuscript or appendix.

### There Was Nothing “Standard” About the “Standard Surgery” Group

Compared with established benchmarks, SANO's “standard surgery” outcomes were surprisingly poor. They report an 84% rate of any complication, 27% rate of anastomotic leak, 10% rate of chylothorax, and an 8% rate of postoperative death (3% at 30 days and 5% at 90 days).^3^ Moreover, salvage esophagectomy—usually a greater-risk procedure—was superior to “standard surgery” in nearly every clinical and technical category, including complications (82% vs 84%), operative duration (304 vs 338 minutes), blood loss (88 vs 100 mL), conversion to open surgery (3% vs 5%), anastomotic leaks (22% vs 27%), reintubation (2% vs 5%), hospital stay (10 vs 11 days), intensive care unit stay (1-2 days vs 1-3 days), and death (30-day mortality 1% vs 3% and 90-day morality 4% vs 5%). This is unusual because salvage esophagectomy outcomes are typically reportedly to be markedly worse.^4^, ^5^, ^6^

Surgery is usually performed between 6 and 8 weeks after chemoradiation to balance tumor responses with operative safety.^1^ Beyond 8 weeks, treatment-related fibrotic change increases technical difficulty and tissue friability, leading to greater rates of infections, anastomotic leaks, and death.^7^, ^8^, ^9^ Salvage esophagectomy—usually performed far later—is known to carry even greater operative risks, although the SANO results appear to suggest the opposite.^4^^,^^5^^,^^10^^,^^11^ Although there are multiple explanations, the best may be that at 12 weeks after chemoradiation, patients who underwent surgery likely underwent esophagectomy at the most dangerous possible time.

The DICE trial (Delayed surgical Intervention after Chemoradiotherapy in Esophageal cancer) provides strong evidence for a 50-day cut-off between standard and salvage surgery on the basis of increasing rates of surgical complications.^6^ If true, this would mean every patient in the SANO trial underwent salvage esophagectomy, which may help explain the poor outcomes.

It is not clear why the authors decided to perform 2 response evaluations and wait 12 weeks after chemoradiation to randomize patients and then perform surgery. There is no existing guideline to serve as precedent for this timing, although there are many sources that would caution against it.^7^, ^8^, ^9^^,^^12^ Without following established modern protocols, it is difficult to draw generalizable conclusions from any other these results.^1^

### Study Design

There are several immediately concerning elements of the study design. After patient crossover from the Pre-SANO trial, there were 111 patients in the surgery arm and 198 in the AS arm; a nearly 2:1 ratio between asymmetric populations.^13^

All forms of esophageal cancer were included, although studies suggest differences in tumor behavior between adenocarcinoma and squamous cell.^14^^,^^15^ There were similar proportions of both adenocarcinoma and squamous cell in both arms (74% and 24% in AS vs 76% and 21% in surgery, respectively), but that simply means the same potential bias was present in both groups.

The primary outcome of overall survival was measured at 2 years—instead of 5—by the interval between CCR and the date of all-cause mortality or last follow up. Unfortunately, median follow-up appeared to differ significantly between the 2 groups. In the AS group, median follow-up was 34 months and in surgery it was 50 months, which likely affected outcomes reporting.

Paradoxically, despite a 3% benefit in 2-year survival with AS, nearly every secondary outcome appeared to favor the surgical group. Median overall survival was 43 months for the AS group and 53 months for surgery. The development of metastases occurred in 43% of patients in the AS group compared with only 34% for surgery. Median follow-up was 34 months (30-40) in the AS group and 50 months (40-60) for surgery. Quality of life (QoL) was found to be significantly improved at 6 and 12 months during AS, likely reflecting postoperative recovery, but there were no differences in QoL after 1 year.

It is also not immediately evident why a stepped-wedge cluster randomization (SWCR) study design was appropriate. SWCR is known to increase the risk of bias compared with parallel cluster randomization.^16^ This vulnerability is acceptable in scenarios in which (1) clusters are relatively homogenous, (2) if researchers believe that there is already sufficient evidence to support their conclusions, or (3) if logistical constraints prevent timely performance of interventions.^17^ Unfortunately, it is not clear how those scenarios can be reasonably applied to a 4-year study evaluating a controversial question on esophageal cancer.

The authors state the SWCR assisted with patients “not understanding the concept of randomization” and to “retain interpretation of a causal effect.” However, it is not evident how SWCR would address either of these concerns.

In fact, SWCR may have adversely affected the standardized delivery of treatment. The median time to surgery was 0.7 months (3 weeks) after the 12 week CRE, making the median time from treatment to surgery 15 weeks. However, there was also significant variability with delays ranging from 12 to 33 weeks after chemoradiation (0.1-4.9 months). Although it remains unclear whether this randomization strategy ultimately resulted in the observed surgical delays, additional clarification would help to provide context for these findings.

### Does it Matter?

The most obvious response to these criticisms is that none of them matter. Even if the CRE protocol is flawed, and even if surgery was performed at the wrong time, patients did better at 2 years if they underwent AS.

But did they? Despite dangerous timing, and an 8% perioperative mortality rate, surgery outperformed the AS group in nearly every category: median survival, median follow-up, local recurrence, and distant metastases. It is therefore surprising that 2-year survival was worse, especially when it relied on patient follow-up, which was longer in the surgical group by 16 months.

All patients who developed recurrence before randomization were excluded. This should not be considered a flaw of study design because the goal was to isolate therapeutic outcomes. However, the data still offer an important perspective. Of the original 776 patients who enrolled, 502 (65%) never made it to randomization. Of the 198 patients selected for AS, 129 (65%) developed recurrence and/or metastatic disease.

As mentioned previously, SANO was successful in identifying a subset of 58 individuals in the AS group (29%) who have not yet developed recurrence, although only 2 have achieved 5-year survival. Perhaps more importantly, without a reliable method to confirm disease-free patients, it will be difficult to apply this observation clinically.

## Conclusions

The SANO trial suggests that definitive chemoradiation has a 10% greater rate of distant metastases, a 16-month shorter median survival, and no long-term benefit to QoL. AS relies on a protocol with a false-negative rate of 65%, possibly identifying the reason for a similar 65% rate of nonsurgical recurrence. Many conclusions of the trial are obscured by poor study design and a methodology that depends on CCR, a dangerous concept that reinforces false confidence and threatens patient safety. Positron emission tomography/computed tomography, endoscopic ultrasound, and endoscopic biopsy are all known to have significant limitations and are not reliable diagnostic tools alone; especially following chemoradiation.

By publishing these outcomes, the authors may have provided the strongest condemnation of definitive chemoradiation in the literature. Physicians should discuss nonsurgical management in some borderline cases, as long as patients are informed of the risks. But without more compelling evidence, esophagectomy should not be abandoned in healthy candidates. Contrary to its conclusions, SANO suggests we should “say no” to AS.

## Conflict of Interest Statement

The authors reported no conflicts of interest.

The *Journal* policy requires editors and reviewers to disclose conflicts of interest and to decline handling or reviewing manuscripts for which they may have a conflict of interest. The editors and reviewers of this article have no conflicts of interest.
